# Elimination of Senescent Cells by Senolytics Facilitates Bony Endplate Microvessel Formation and Mitigates Disc Degeneration in Aged Mice

**DOI:** 10.3389/fcell.2022.853688

**Published:** 2022-07-08

**Authors:** Bolin Chen, Runjiu Zhu, Hao Hu, Mingbin Zhan, Tingxuan Wang, Fangli Huang, Fuxin Wei, Yu Chai, Zemin Ling, Xuenong Zou

**Affiliations:** ^1^ Guangdong Provincial Key Laboratory of Orthopedics and Traumatology, Department of Spinal Surgery, The First Affiliated Hospital of Sun Yat-sen University, Guangzhou, China; ^2^ Division of Orthopaedics and Traumatology, Department of Orthopaedics, Nanfang Hospital, Southern Medical University, Guangzhou, China; ^3^ Department of Orthopedics, The Seventh Affiliated Hospital of Sun Yat-sen University, Shenzhen, China

**Keywords:** intervertebral disc degeneration, aging, senescence, senolytics, bony endplate

## Abstract

Senolytics are a class of drugs that selectively eliminate senescent cells and ameliorate senescence-associated disease. Studies have demonstrated the accumulation of senescent disc cells and the production of senescence-associated secretory phenotype decrease the number of functional cells in degenerative tissue. It has been determined that clearance of senescent cell by senolytics rejuvenates various cell types in several human organs, including the largest avascular structure, intervertebral disc (IVD). The microvasculature in the marrow space of bony endplate (BEP) are the structural foundation of nutrient exchange in the IVD, but to date, the anti-senescence effects of senolytics on senescent vascular endothelial cells in the endplate subchondral vasculature remains unclear. In this study, the relationships between endothelial cellular senescence in the marrow space of the BEP and IVD degeneration were investigated using the aged mice model. Immunofluorescence staining was used to evaluate the protein expression of P16, P21, and EMCN in vascular endothelial cells. Senescence-associated β-galactosidase staining was used to investigate the senescence of vascular endothelial cells. Meanwhile, the effects of senolytics on cellular senescence of human umbilical vein endothelial cells were investigated using a cell culture model. Preliminary results showed that senolytics alleviate endothelial cellular senescence in the marrow space of BEP as evidenced by reduced senescence-associated secretory phenotype. In the aged mice model, we found decreased height of IVD accompanied by vertebral bone mass loss and obvious changes to the endplate subchondral vasculature, which may lead to the decrease in nutrition transport into IVD. These findings may provide evidence that senolytics can eliminate the senescent cells and facilitate microvascular formation in the marrow space of the BEP. Targeting senescent cellular clearance mechanism to increase nutrient supply to the avascular disc suggests a potential treatment value of senolytics for IVD degenerative diseases.

## 1 Introduction

Intervertebral disc degeneration (IDD) has been accepted as a primary cause of low back pain, LBP (Smith et al., 2011), which is ranked as one of the top causes of years lived with disability (Buser et al., 2019). In addition to LBP, IDD can also be secondary to a series of spinal degenerative diseases. Once it is advanced, the options of clinical interventions are admittedly limited, and the patients usually require surgery interventions in the late stage of IDD (Wenger and Cifu, 2017; Kamali et al., 2021). It gives rise to an urgent need to understand the underlying mechanisms of disc degeneration and develop solutions to delay or ameliorate the age-dependent progression of intervertebral disc (IVD), especially in the early or middle period of IDD (Vergroesen et al., 2015; Vo et al., 2016).

As research continues, it is now recognized that aging exacerbates disc degeneration and disease progression (Vo et al., 2013; van Deursen, 2014). In recent years, researchers have identified the senescent cells during IVD aging and degeneration, which is characterized by cell cycle arrest and the production of catabolic factors known as the senescence-associated secretory phenotype (SASP) (Wang et al., 2016; Novais et al., 2019). These specific cell phenotypes were found to be induced in response to a variety of adverse stimulation and accumulated in degenerated IVDs with apoptotic resistance by activating relevant signaling pathways. Previous studies have revealed their numbers increase in aged animals and humans, which demonstrates that crucial roles of cellular senescence in the initiation and development of IDD (Ngo et al., 2017; Yamane et al., 2020). Meanwhile, a large class of drugs that can induce apoptosis in senescent cells, allowing the remaining non-senescent population to preserve or restore tissue function, have created greater interest for their ability of selectively eliminating senescent cells (Kirkland et al., 2017; Cherif et al., 2020). Dasatinib (D), a Src/tyrosine kinase inhibitor, and quercetin (Q), a natural flavonoid that binds to BCL-2 and modulates cell cycle proteins, are an extensively representative drug class, termed senolytics. It has been reported that dasatinib can clear senescent adipocyte progenitor cells, while quercetin can kill senescent endothelial cells (ECs) and osteoblast (Zhu et al., 2015). As a result, the combination of dasatinib and quercetin also has a promising prospect in clinical use for the potential therapeutic effect on improving physical condition and life span (Xu et al., 2018). A recent study by Novais et al. (2021) revealed that long-term treatment with targeting cellular senescence has a stronger effect on preventing age-related disc degeneration in mice. While these studies implicate senescent cells in driving disc degeneration, it is still a key issue to confirm that the elimination of senescent cell has the great promoting effect on alleviating multiple senescence-related phenotypes in IVD and takes it as the priority therapeutic target.

With the increase of age, the function and interaction of three unique IVD compartments: the central nucleus pulposus (NP), the circumferential annulus fibrosus (AF), and the cranial and caudal cartilaginous endplates (CEP) continue to deteriorate, which is difficult to avoid (Roberts et al., 2006; Sharifi et al., 2015). Importantly, normal IVD is the largest avascular structure in human body and exchanges metabolites via diffusion from the adjacent capillary bed penetrating the subchondral bone of the endplate and the capillaries around the fibrous ring. The main nutrient supply of the IVD comes from the bony endplate vasculature, and material exchange between the vertebral body and the IVD is carried out through the diffusion of the cartilaginous endplate, which is the nutrient supply route of the disc cells (Urban et al., 2004; Gullbrand et al., 2014). As life span increases, so does the cartilaginous endplate osteosclerosis changes, accompany with the number of microvessels under the bony endplate gradually decreases, and the permeability disappears, resulting in the imbalance of energy metabolism of nucleus pulposus cells (Benneker et al., 2005; Ashinsky et al., 2020). Consequently, microvessels under the bony endplate and nutrient availability at the bone–disc interface decreases may be a key factor of IDD that could not be neglected. During IVD degenerative process, there are inevitable interactions between the human IVD cells and adjacent non-IVD cells, including ECs which play a major part in vascular structure formation (Hwang et al., 2020). The senescent vascular endothelial cellular accumulation, which leads to the altered cellularity, vascular regression, and extracellular matrix composition, might set the IVD on a slow course toward degeneration. Additionally, the positive association of the vascular endothelial cellular senescence with the decrease of microvasculature in the marrow space of the bony endplate, which can hinder transport from nutrient supply to the disc or result in changes in cell phenotype, even death. In consideration of the results of our previous study showing that degenerative changes in the endplate, which blocked the nutrient diffusion from vessel network under the bony endplate, occurred earlier than the nucleus pulposus degeneration (Zhong et al., 2016; Ling et al., 2020), we wonder whether cellular senescence in development of vascular aging phenotypes under bony endplate involves in the mechanism of preventing disc degeneration by senolytics.

In this study, we aimed to investigate whether the elimination of senescent cells by senolytics (dasatinib and quercetin) could prevent microvascular changes in the marrow space of bony endplate, and in particular, endothelial cell senescence phenotypes in the aged mouse model. We also studied in vitro the indirect effects of senolytics on facilitating bony endplate microvessel formation, which provides important evidence that senescent endoepithelial cells play a significant role in the progression of IDD and senolytics have a promising prospect as an effective clinical therapeutic in age-dependent disc degeneration in the future.

## 2 Materials and Methods

### 2.1 Animal Model and Design

**FIGURE 1:**
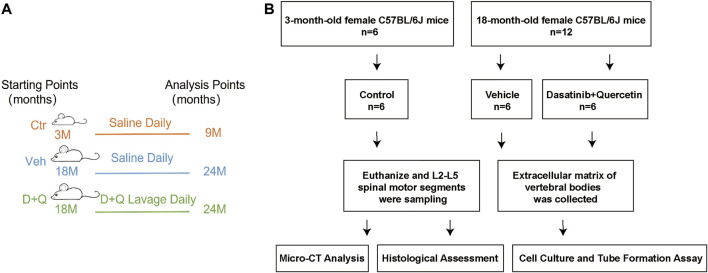
Overall experimental design. **(A)** Schematic showing research time points of aged mice model in this study. **(B)** Experimental flow chart. Micro-CT, microcomputed tomography; Ctr, control group (3-month-old mice, saline); Veh, vehicle group (18-month-old mice, saline); D + Q, dasatinib and quercetin group (18-month-old mice, 5 mg/kg/day dasatinib plus 50 mg/kg/day quercetin).

We purchased the female mice (C57BL/6J background) from the Animal Center of Sun Yat-sen University (Guangzhou, China) at 3 months and 18 months of age. Administrative Committee of the Experimental Animal Care and Use of Sun Yat-sen University (Approval No. 2021000259) authorized the animal study. Pathogen-free mice were maintained under regulated settings of temperature (22–28°C) and relative humidity (40–60%) with day and night rotations. The mice were housed in mouse small shoebox cages with freely available food and tap water as well. Eighteen-month-old mice were randomly allocated into two equal groups (six in one cage) after a 1-week adaption period: vehicle group and dasatinib and quercetin group. Three-month-old mice (six in one cage) were selected randomly as a reference: control group. According to the procedure established previously (Su et al., 2020), dasatinib (S1021, Selleck Chemicals, Houston, TX, United States) and quercetin (S2391, Selleck Chemicals, Houston, TX, United States) were diluted in 10% PEG400 and delivered to the dasatinib and quercetin group by oral gavage at dosages of 5 and 50 mg/kg/day, respectively. The control and vehicle groups were treated with the same amount of normal saline daily and their food intakes were matched as well. The animals were euthanized at the day after receiving a 6-month gavage, and pentobarbital sodium (90 mg/kg, i.p.) anesthesia was used during the procedure. The L2–L5 spinal motion segments were dissected and collected for future experiments (Figure 1).

### 2.2 Microcomputed Tomography Scanning and Analysis

The L2–L5 vertebrae were dissected free of soft tissue from mice, analyzed by using a desktop Micro-CT SkyScan1276 (Bruker Micro CT, Belgium) after fixing with 4% paraformaldehyde for 24 h. The scanner was set at a voltage of 85 kVp and a current of 200 μA with a spatial scanning resolution of 6.8 μm per pixel. Aluminum (1-mm thick) filter was used for optimal image contrast. The order of the vertebrae from L2 to L5 was identified based on the ribs on the lower thoracic. Image reconstruction software (NRecon v1.6), data analysis software (CTAn v1.9), and three-dimensional model visualization software (CTVol v2.0) were used to analyze the parameters of L2–L5 vertebrae. L3 and L4 were separated from cortical bone by free-drawing region of interest (ROI). Volume of interest (VOI, L3 and L4 vertebral bodies) was chosen within 80 continuous slices. Morphologic measurements were performed in CTAn and the trabecular bone volume fraction (BV/TV; %) was obtained.

The scanner was set at a voltage of 49 kVp, a current of 200 μA, and a resolution of 6.8 μm per pixel to measure the L3/4 IVD. Coronal images of the L3/4 IVD were used to perform three-dimensional histomorphometric analyses of IVD. IVD volume was defined by the ROI to cover the whole invisible space between L3 and L4 vertebrae. EP was defined by a free-drawing ROI that was positioned over the visible bony plate close to the vertebral bodies, separated by the growth plates. The bone volume (BV)/total volume (TV), trabecular number (Tb.N; 1/mm), trabecular pattern factor (Tb.pf; mm), trabecular separation (Tb.Sp; mm), and structural model index (SMI) were calculated by assigning a threshold content within 80 continuous slices through software CTAn. Then, three-dimensional models of volume of interest (VOI) were reconstructed with model visualization software, CTVol for morphologic measurements. The operator conducting the micro-CT analysis was blinded to the treatments associated with samples.

### 2.3 Histological Assessment

The L2–L5 spinal motion segments fixed with 4% neutral paraformaldehyde for 72 h were decalcified in 0.5 M ethylene diamine tetraacetic acid (EDTA, pH 7.4) for 7 days at room temperature, followed by paraffin embedding or frozen embedding. The coronal surfaces of decalcified L2–L5 vertebrae were sectioned longitudinally in 4-µm-thick sections. According to standard procedures, hematoxylin–eosin (H&E) staining, safranin O-fast green staining, immunofluorescence staining, and senescence-associated β-galactosidase staining were performed and the number of positive stained cells per square millimeter (N. per mm^2^) was measured in the area from 0 to 0.2 mm below bony endplate.

#### 2.3.1 H&E and Safranin O Staining

Paraffin tissue slices were deparaffinized and hydrated to distilled water. Then, H&E and safranin O-fast green were guided by the instructions of the reagent kits (Servicebio Biological Technology Co., Ltd., China). H&E staining was used to well distinguish bone tissue structure and cells in bone marrow cavity as well as determine the disc construction. After safranin O-fast green staining, the cartilage and mucin will be stained orange to red and the nuclei will be stained black, which was performed for the analysis of IVD microstructure. The images were observed and captured by an image scanning microscope (Leica, Germany). And then, we applied a quantitative mouse IVD histopathological scoring system to evaluate the IDD from safranin O-fast green sections. The scoring system could analyze key histopathological features including NP, AF, EP, and AF/NP/EP boundary regions. Each image was scored independently by two blinded raters, and the average score was used for further analysis.

#### 2.3.2 Immunofluorescence Staining

Paraffin tissue slices were rehydrated, then antigens were repaired using EDTA-Tris (70°C, 90 min). After washing with PBS three times, antigens were closed in 10% goat serum for 1 h and incubated with primary antibodies against P16 (1:100, Abcam ab211542), P21 (1:100, Proteintech 28248), Ki67 (1:200, Abcam ab15580), OCN (1:200, Proteintech 23418), EMCN (1:500, Santa Cruz Biotechnology), and VEGF (1:500, Affinity AF5131) at 4°C for overnight. Tissue sections were washed with PBST three times next day and incubated with Alexa Fluor plus 488 goat anti-rat (1:500 Thermo Fisher Scientific A48262) or Alexa Fluor plus 594 goat anti-rabbit (1:500 Thermo Fisher Scientific A32740) at room temperature for 1 h. After washing samples with PBST three times for 5 min each, nuclei were counterstained with DAPI (S2110, Solarbio, China). Sections were visualized with an Axio Imager 2 microscope using a 5/0.15 N-Achroplan or 20/0.5 EC Plan Neofluar objective and caught on an Axiocam MRm monochrome camera (Carl Zeiss) using of Zen2TM software (Carl Zeiss AG, Germany). ImageJ software v1 was used to quantify the number of positive stained cells per square millimeter of the endplate subchondral bone region (N. per mm^2^), which was measured from 0 to 0.2 mm below bony endplate. Images containing chosen ROIs were established the threshold limit value to remove background, converted to binary format, and then stained cell numbers were determined by using analysis particle function of ImageJ software v1.

#### 2.3.3 Senescence-Associated β-Galactosidase Staining

Frozen tissue slices were stained using SA-β-Gal staining kit (Solarbio, G1580) according to the manufacturer’s instructions. In brief, frozen sections were thawed for 30 min at room temperature. After washing the tissue with PBS for three times, we applied the required amount of SA-β-Gal fixative to cover the tissue completely and left it to cure for at least 15 min at room temperature. Then, rinsed the tissue three times in PBS, each time for at least 5 min. The staining working solution was set up according to the directions’ ratio and incubated for 8 h at 37°C. After staining, senescent cells were identified as blue-stained cells under a light microscope. Image Pro Plus was used to catch the area from 0 to 0.2 mm below bony endplate and quantify the number of blue-stained cells per square millimeter (N. per mm^2^).

### 2.4 Extracellular Matrix of Vertebral Body Collection

The lumbar vertebral bodies (VBs) were dissected from the L2 to –L5 spinal motion segments of 3- and 18-month-old female mice, which were treated with either dasatinib and quercetin dose or vehicle, and were removed under sterile conditions. The collected L2–L5 VBs were rinsed with the Dulbecco’s modified Eagle’s medium (DMEM, Invitrogen, Carlsbad, CA, United States) supplemented with 1% penicillin–streptomycin (MediaTech, Dallas, TX, United States) and 10% lot-selected fetal bovine serum (FBS, Atlanta Biologicals), and the cell supernatant had been collected after centrifugation at 2,000 *g* for 10 min. The combination of equal parts of cell supernatant and DMEM supplemented with 1% penicillin–streptomycin and 10% FBS is the extracellular matrix of vertebral bodies (ECM VBs) from Ctr, Veh, and D + Q groups. The ECM VBs were stored at −80°C for use of downstream experience.

### 2.5 Cell Culture and Tube Formation Assay

Cryopreserved human umbilical vein endothelial cells (HUVECs) were purchased from ATCC (PCS-100-013, Gaithersburg, MD). Matrigel (BD Biosciences) was plated in 96-well culture plates and incubated at 37°C to polymerize for 45 min. Then, HUVECs were seeded onto Matrigel-coated 96-well plates at a density of 1 × 10^5^ cells/well and the cell culture medium was changed into DMEM supplemented with 1% penicillin–streptomycin and 10% FBS, or individual ECM VBs medium prepared as described earlier and cultured for 24 h. Thus, HUVECs, a cell line with tube formation, were exposed to conditioned medium (CM) collected from Ctr, Veh, and D + Q groups. Tube formation was observed under the microscopy, and the cumulative tube lengths and loops were measured after incubation at 37°C for 6 h.

### 2.6 Statistical Analysis

Statistical analysis complied with the recommendations on experimental design and analysis in pharmacology and was performed using Prism software (version 6.0.1). For all data comparisons, Shapiro–Wilk normality and homogeneity of variance tests were performed and quantitative data were represented as mean ± standard deviation. For comparisons between three groups’ quantitative data, we used one-way analysis of variance with post hoc Tukey’s honest significant difference test after the evaluation of normal distribution and homogeneity of variance. In all analyses, when *p* values were below 0.05, differences were considered statistically significant. The experiment was randomized, and the assignment was blind during the experiment and result analysis. The same sample is not measured repeatedly. The reported results were consistently repeated across multiple experiments.

## 3 Results

### 3.1 D + Q Treatment Increases Vertebral Bone Mass and Intervertebral Disc Volume in Aged Mice

**FIGURE 2:**
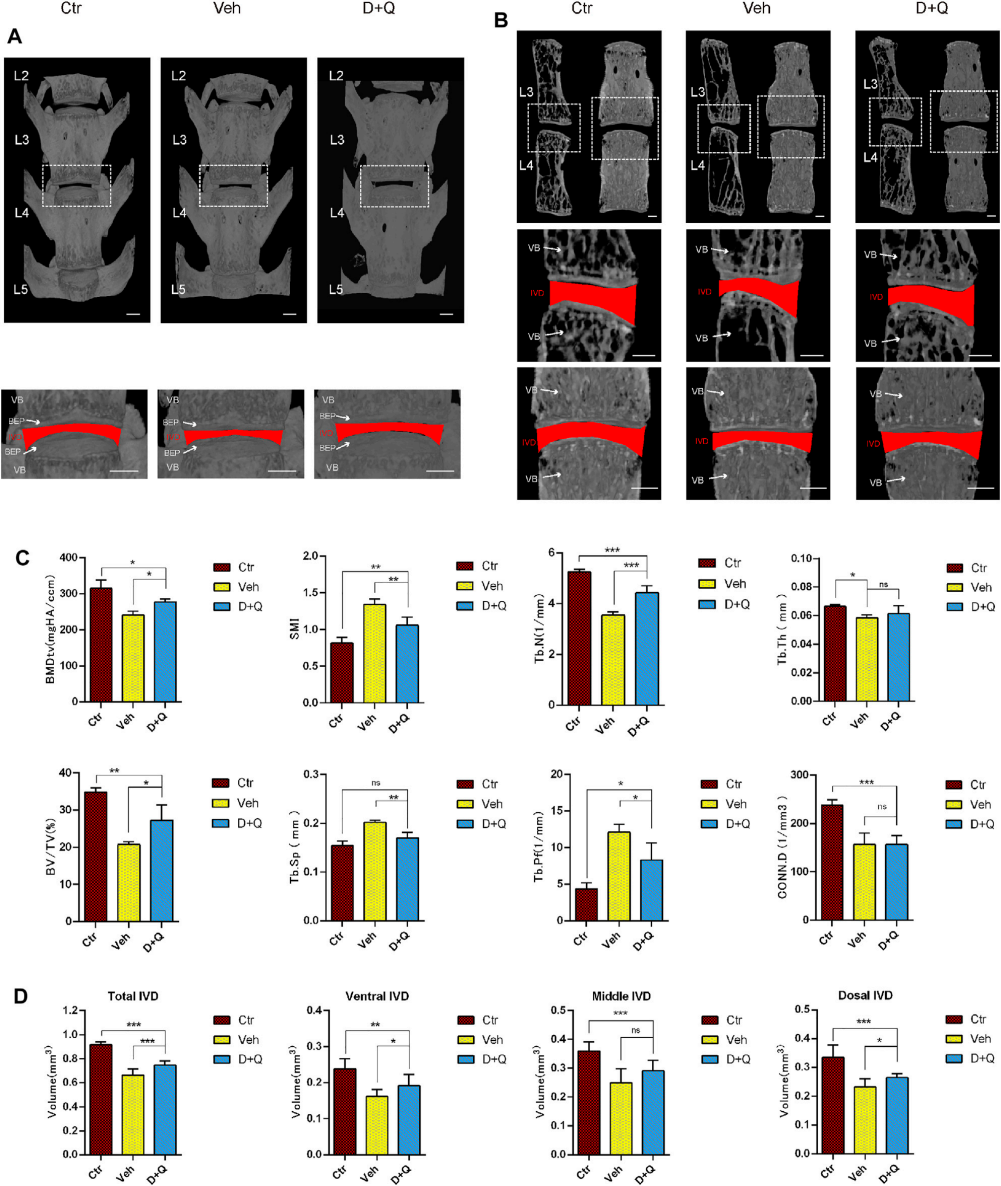
Changes in L2–L5 microarchitecture parameters of the intervertebral disc and vertebral bone determined by microcomputed tomography. **(A)** Coronal view of microcomputed tomography reconstructed image of L2–L5 vertebrae bone. The white dotted box is zoomed in, as coronal view of the L3/4 intervertebral disc. The red area represents the disc volume. **(B)** Coronal and sagittal section view of L3 and L4 vertebrae bone. The white dotted box is zoomed in, as coronal view of the L3/4 intervertebral disc. The red area represents the disc volume. **(C)** The bone morphometry parameters of L2–L5 trabecular bone. Markedly increased bone volume (BV)/total volume (TV), trabecular number (Tb.N; 1/mm), and decreased trabecular pattern factor (Tb.pf; mm), trabecular separation (Tb.Sp (mm), and structural model index (SMI) in the D + Q group are shown when compared with the control group. **(D)** The quantified data of L3/4 intervertebral disc volume. Markedly increased volume of intervertebral disc in the D + Q group is shown when compared with the control group. EP, endplate; Ctr, control group; Veh, vehicle group; D + Q, dasatinib and quercetin group. The ROI of the disc is indicated by the red color. Significance was determined using one-way analysis of variance with post hoc Tukey’s honest significant difference test. D + Q vs. Ctr group and Veh group; ns, no significance; *p < 0.05; **p < 0.01; ***p < 0.001.

Previous studies have reported that the clearance of senescent cells using senolytics show a positive effect on improving bone mass and inhibiting IDD (Farr et al., 2017; Lim et al., 2022). To determine the effectiveness of the dasatinib and quercetin to attenuate an age-dependent progression of disc degeneration, aged mice were given dasatinib and quercetin through daily oral gavage for 6 months and then were sacrificed for Micro-CT measurement. The characteristics of vertebrae and IVD in each group were measured and compared by micro-CT image reconstruction (Figure 2A). The bone trabeculae in the vehicle group were sparser than the other two groups accompanied with IVD height loss (Figure 2B). Similarly, the results of quantitative examination of vertebrae trabecular formations revealed that the vehicle group exhibited a significant reduction compared to the control group as well as the D + Q group (Figure 2C). The parameter analysis of microarchitecture of vertebral body indicates a decrease in vertebral bone mass in the vehicle group and an increase in the D + Q group. In addition, markedly increased bone volume (BV)/total volume (TV), trabecular number (Tb.N; 1/mm), and decreased trabecular separation (Tb.Sp (mm), trabecular pattern factor (Tb.pf; mm), and structural model index (SMI) in the D + Q group are shown when compared with the vehicle group. In addition, progressively narrowed IVD space is the major characteristics of age-related IDD. As can be seen in representative view of reconstructive IVD volume (Figure 2B), the D + Q group showed generally higher IVD volume and height of IVD space. The quantity analysis of the IVD volume (Figure 2D) indicated that the effective increase especially occurred in ventral and dorsal IVD. These results showed that the vertebral bone mass and IVD volume decreased with age, which could be reversed by the D + Q senolytics treatment.

### 3.2 D + Q Treatment Ameliorates IDD by Restoring IVD Structure

**FIGURE 3:**
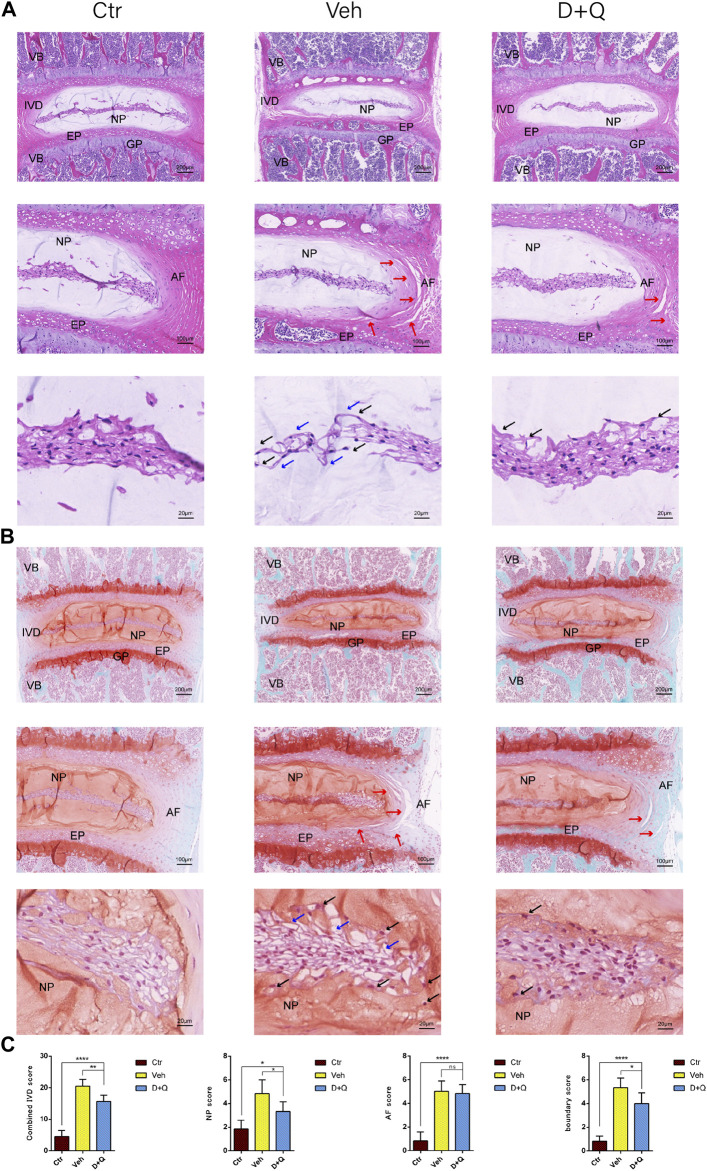
Histological evaluation of intervertebral disc degeneration. **(A)** Coronal sections of IVD stained by hematoxylin and eosin in Ctr, Veh, and D + Q groups. Panoramic images of IVD pathology and higher magnification of the annulus fibrosus (AF), endplate (EP), and nucleus pulposus (NP). Increased number of traps in AF (red arrow), rare fibrous located between NP cells (blue arrow) and cluster of small and rounder NP cells (black arrow). **(B)** Coronal sections of IVD and endplate stained by safranin O-fast green in Ctr, Veh, and D + Q groups. Red indicates proteoglycan and green stains calcified cavities. Panoramic images of IVD pathology and higher magnification of the AF, EP, and NP. Loss of NP-AF boundary (red arrow), rare fibrous located between NP cells (blue arrow) and cluster of small and rounder NP cells (black arrow). **(C)** Combined IVD score, NP score, AF score, and AF/NP/EP boundary score of Ctr, Veh, and D + Q groups. IVD, intervertebral disc; EP, endplate; Ctr, control group; Veh, vehicle group; D + Q, dasatinib and quercetin group; scale bar = 200 μm (upper), 100 μm (middle), 50 μm (lower); D + Q vs. Ctr group and Veh group; ns, no significance; *p < 0.05; **p < 0.01; ***p < 0.001; ****p < 0.0001.

For further research, a histology investigation was conducted to examine whether dasatinib and quercetin was linked to the maintenance of the normal microstructure of IVD. In comparison to the control group, H&E staining (Figure 3A) showed widening and disorganization of AF lamellae in the vehicle group. The AF of the vehicle group generated an increased number of traps, higher collagen disorganization, and fewer cells. And rare fibrous stands between NP cells were noted, resulting to cluster of small and rounder NP cells. Safranin O-fast green staining (Figure 3B) revealed mild loss of NP-AF boundary with inward fibrosis and merger of AF into NP in the vehicle group, while abnormal IVD height was apparent. In addition, the D + Q group revealed more stellate or spindle-shaped normal NP cells and less rounder cells than the vehicle group. It suggested that in the D + Q group, signatures of IDD were also found, but it was greatly slighter than the vehicle group. To evaluate the whole degenerative IVD by quantitative method, mouse IVD histopathological scoring system previously described (Melgoza et al., 2021) was used to score the NP, AF, and AF/NP/EP boundary regions in every single IVD of each group (Figure 3C). The results showed that in spite of no difference in AF, the vehicle group generally had a significant higher score than the control group, while the D + Q group scored slightly lower. Together, these findings indicate that the degeneration of the whole IVD progressed with age, especially in the NP regions, which could be mitigated by dasatinib and quercetin treatment.

### 3.3 D + Q Treatment Targets and Reduces the Number of Senescent Cells in the Marrow Space of the Bony Endplate

**FIGURE 4:**
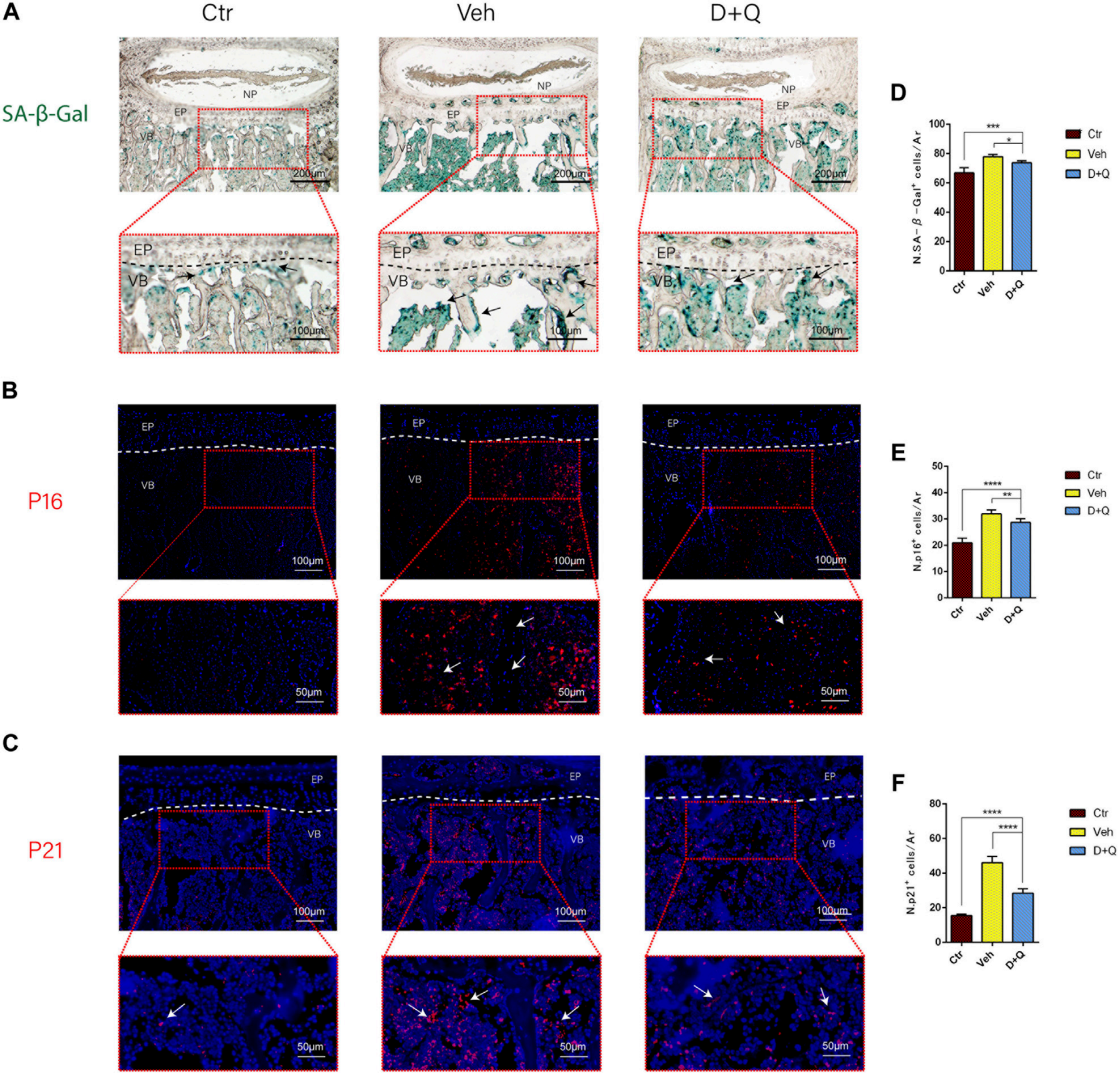
Qualitative and quantitative detection of senescent cells in the sub-endplate region. The expression of **(A)** SA-β-gal, **(B)** P16, and **(C)** P21 could be observed in the intervertebral disc (IVD) by histological assessment. Cell nuclei **(B, C)** were stained with DAPI (blue). **(D-F)** Quantitation of the number of SA-β-gal, P16, and P21 positive cells in the sub-endplate region. Data are represented as mean ± SD. NP, nucleus pulposus; AF, annulus fibrosus; EP, endplate; Ctr, control group; Veh, vehicle group; D + Q, dasatinib and quercetin group. Scale bar = 100 μm (upper), 50 μm (lower). D + Q vs. Ctr group and Veh group; ns, no significance; *p < 0.05; **p < 0.01; ***p < 0.001; ****p < 0.0001.

It had been reported that senolytics could eliminate senescent cells and increase the number of proliferating cells in cultures from degenerated IVD (Cherif et al., 2019). Given that senescent ECs may have a role in age-associated IVD. Our intent was to study the ability of dasatinib and quercetin to eliminate the senescent ECs. The expression of SA-β-Gal and P16, P21 in tissue was detected using SA-β-Gal staining and immunofluorescence staining assay. We first employed SA-β-Gal staining for frozen sections, which revealed that in the vehicle group, a considerable number of blue-stained cells formed in the marrow space of the bony endplate (Figure 4A). In the same space, the accumulation of senescent cells existed, whereas the D + Q group had a smaller accumulation of these cells. Preliminary results showed that in the vehicle group, there was a significant increase in accumulation of senescent cells in the region, but D + Q could clear part of the positive cells. D + Q appeared to have an inhibitory impact on cellular senescence in the marrow space of the bony endplate. To further study cellular senescence in the degenerated IVD, we used immunofluorescence labeling for senescence markers (P16 and P21) in tissue staining. P16 marker was expressed in the IVDs of all three groups, mainly in IVDs of the vehicle group (Figure 4B). P21 marker was shown to be expressed in the vehicle and D + Q groups and was nonexistent in the control group (Figure 4C). The quantified data showed that compared to the vehicle group, the expression of P16 and P21 were both lower in the control and D + Q groups. As a result, these findings implied that the combination of dasatinib and quercetin can partially eliminate senescent cell in the marrow space of the bony endplate to halt the progress of senescence and extracellular matrix dysfunction with age.

### 3.4 D + Q Treatment Increases the Number of Vascular Endothelial and Osteoblast Cells and Facilitates Bony Endplate Microvessel Formation

**FIGURE 5:**
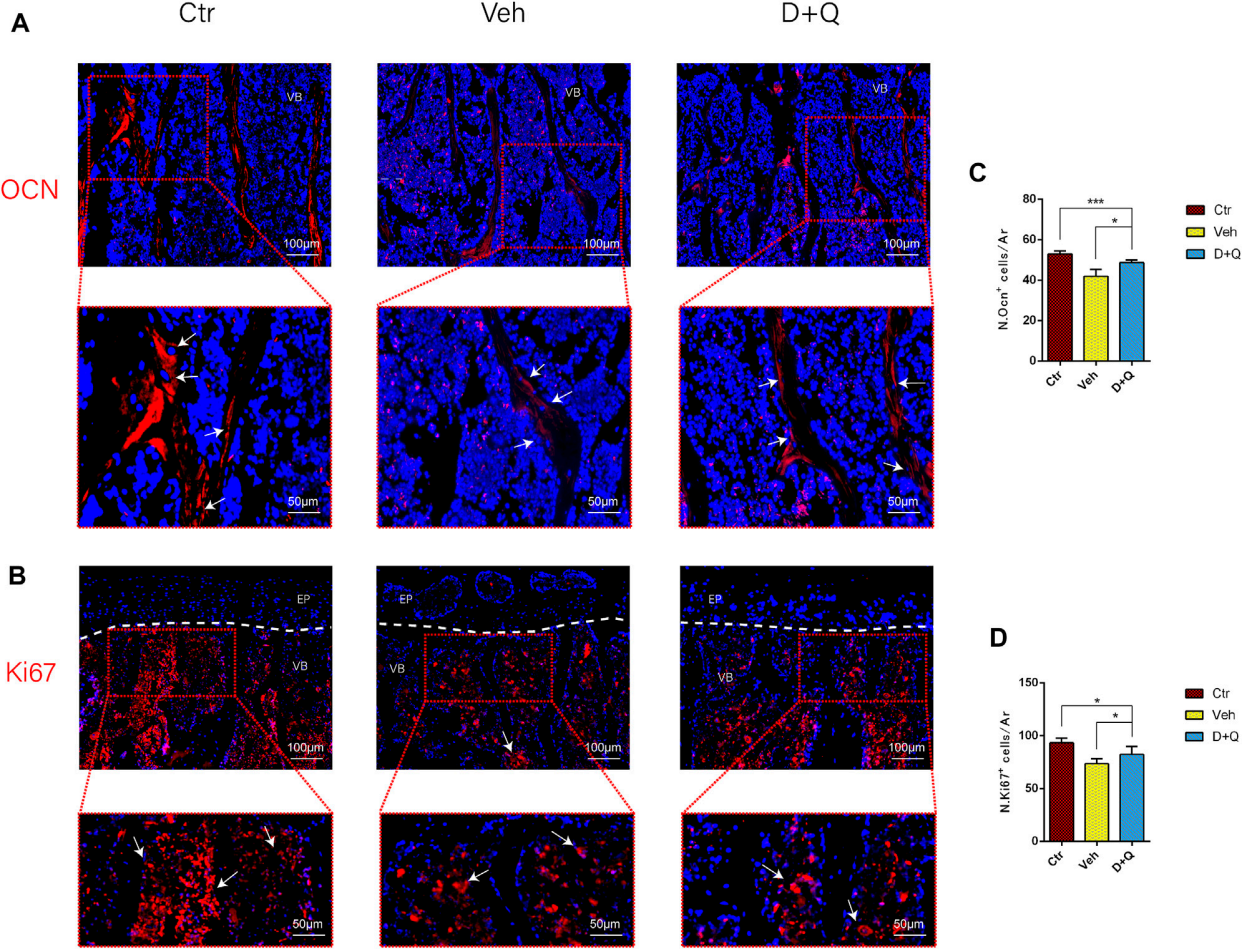
Qualitative and quantitative detection of osteogenic and proliferative marker in the sub-endplate region. The expression of **(A)** OCN and **(B)** Ki67, which could be observed in the sub-endplate region by histological assessment. Cell nuclei were stained with DAPI (blue). **(C**,**D)** Quantitation of the number of OCN and Ki67 cells in the sub-endplate region. Data are represented as mean ± SD. NP, nucleus pulposus; AF, annulus fibrosus; EP, endplate; Ctr, control group; Veh, vehicle group; D + Q, dasatinib and quercetin group. Scale bar = 100 μm (upper), 50 μm (lower); D + Q vs. Ctr group and Veh group; ns, no significance; *p < 0.05; **p < 0.01; ***p < 0.001; ****p < 0.0001.

**FIGURE 6:**
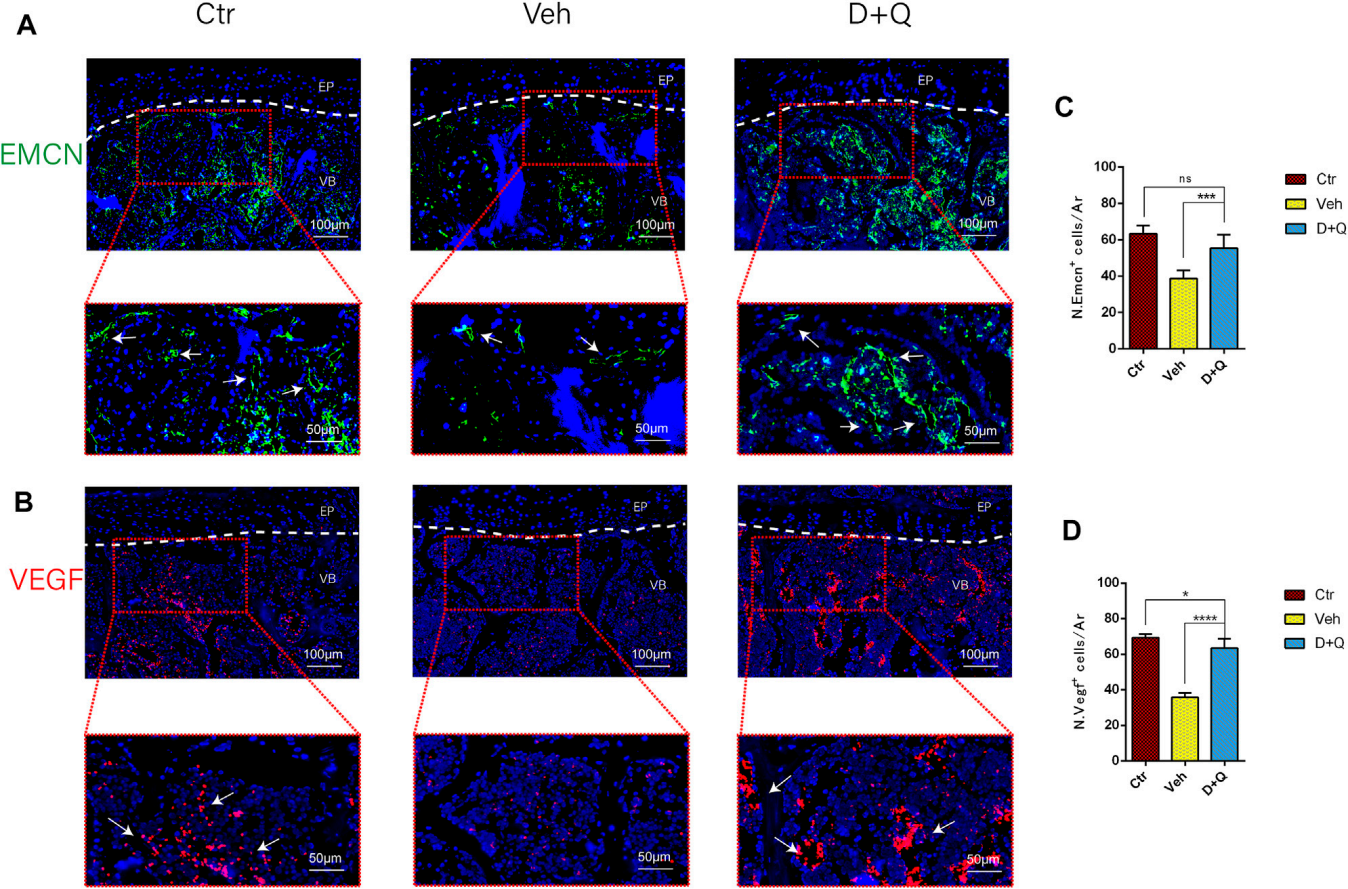
Qualitative and quantitative detection of endothelial and angiogenic marker in the sub-endplate region. Immunofluorescence staining of vascular endothelial cell marker **(A)** EMCN and **(B)** VEGF could be observed in the coronal sections of intervertebral disc (IVD). Cell nuclei were stained with DAPI (blue). **(C,D)** Quantitative data of the EMCN and VEGF cells in the marrow space of bony endplate. Data are represented as mean ± SD. NP, nucleus pulposus; AF, annulus fibrosus; EP, endplate; Ctr, control group; Veh, vehicle group; D + Q, dasatinib and quercetin group. Scale bar = 100 μm (upper), 50 μm (lower); D + Q vs. Ctr group and Veh group; ns, no significance; *p < 0.05; **p < 0.01; ***p < 0.001; ****p < 0.0001.

**FIGURE 7:**
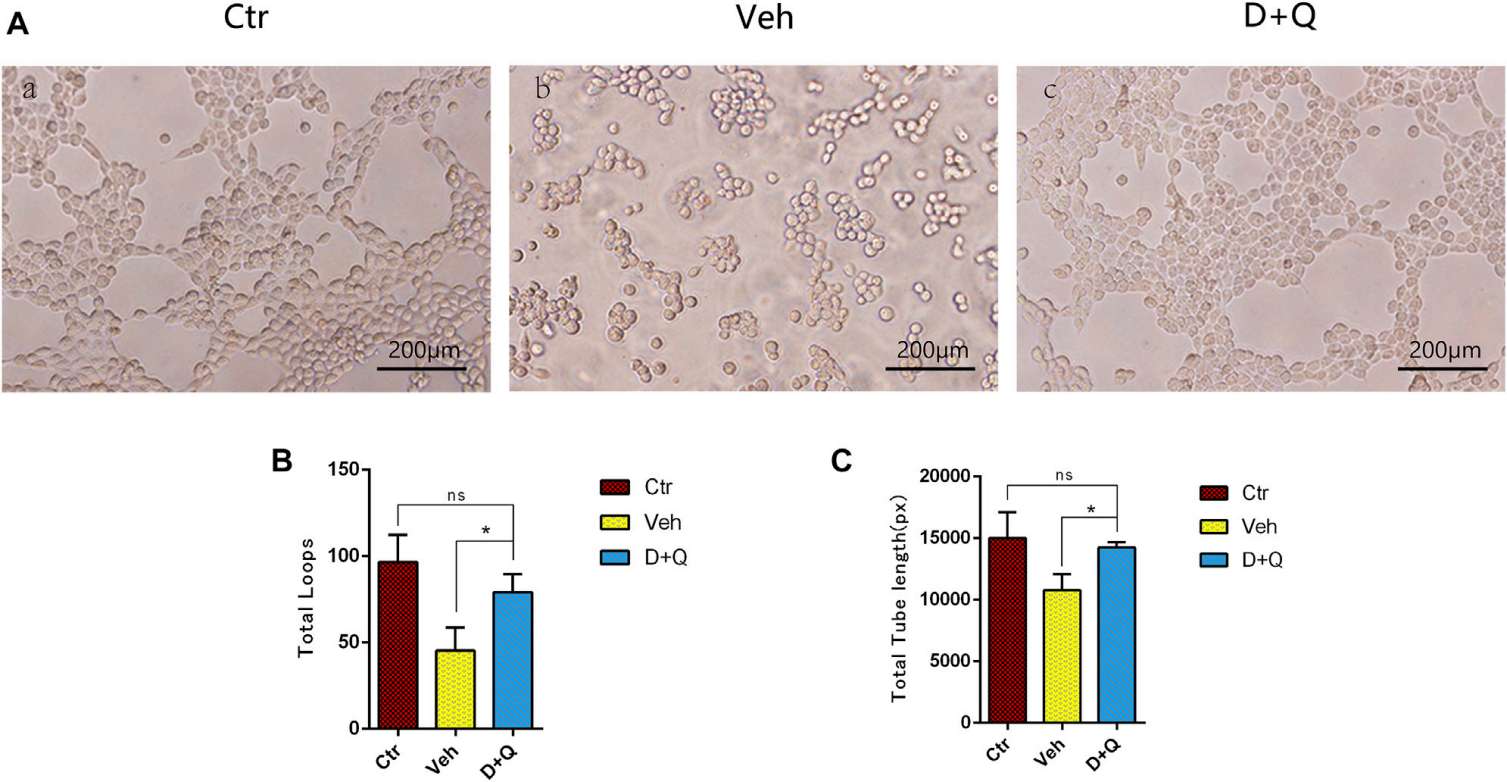
The effect of extracellular matrix of vertebral bodies (ECM VBs) medium on the tube formation of HUVECs. **(A)** Microscopy image shows capillary-like structures and a branching network after 6 h of exposure to the ECM VBs from Ctr group (left). Capillary-like structures and branching network formation are suppressed when exposed to the ECM VBs from Veh group (middle). D + Q exposure gets back to form capillary-like structures and looped branching networks after the 6 h culture period (right). Scale bar = 200 μm. **(B,C)** Quantitative data of loops and length of tubes. Data are represented as mean ± SD. NP, nucleus pulposus; AF, annulus fibrosus; EP, endplate; Ctr, control group; Veh, vehicle group; D + Q, dasatinib and quercetin group. D + Q vs. Ctr group and Veh group; ns, no significance; *p < 0.05.

To further evaluate the role of decreased cellular senescence in IDD and bone metabolism, we performed staining of osteogenic marker OCN, proliferative marker Ki67, and vessel marker EMCN and VEGF, respectively. Immunofluorescence staining of OCN and Ki67 (Figure 5A) revealed that a large number of mature osteoblasts accumulated in the vertebral body in the control group where OCN positive cells were detected intensively. Instead, positive cells number in the vehicle group was significantly lower than the control group, but the D + Q group had a considerable improvement. Additionally, D + Q treatment also partly restored the number of Ki67 positive cells compared to the vehicle group (Figure 5B). According to quantitative results (Figures 5C,D), it is suggested that D + Q reduces growth arrest and senescence in certain cell types. In addition, a large number of EMCN staining positive cells and VEGF positive cells were present in the marrow space of the bony endplate in the control and D + Q groups (Figures 6A,B), while the number of positive cells in the vehicle group was much less. Quantitative immunofluorescence analyses showed increases in EMCN and VEGF in both Ctr and D+Q groups relative to the Veh group (Figures 6C,D). The similar expression patterns of Ki67, EMCN, and VEGF suggest that the clearance of senescent cells may contribute to the proliferation of newborn ECs and osteoblasts, which promote the coupling of angiogenesis and osteogenesis in the vertebral bone. To further confirm this, HUVECs, a cell line with tube formation ability, were exposed to individual extracellular matrix of vertebral bodies (ECM VBs) medium collected from the control, vehicle, and D + Q groups, respectively. The results showed that the ECM VBs of the vehicle group produced adverse effects on the angiogenesis of HUVECs (Figure 7A) compared with other two groups. Indeed, D + Q ECM VBs treatment can eliminate these adverse effects on HUVECs and markedly improve their ability of tube formation. Meanwhile, it could be observed that the tube structure of the D + Q group was richer than that of other two groups. The length and loops of tubes, which simulated sprouting of vascular, was calculated more in D + Q groups (Figures 7B,C). Given the above findings, D + Q appears to have a stimulative impact on the formation of newborn microvessels under bony endplate and subsequently improve the nutrient supply to the adjacent IVD and vertebral bone.

## 4 Discussion

Degeneration of the IVD is one of the major causes of LBP. Yet there is currently no effective therapy for IDD, and spine fusion surgery is the only option for patients in the late stage of IDD (Dowdell et al., 2017). As a result, to find a treatment that can slow or stop the progression of IDD is critical and meaningful. Since the underlying mechanism involved IDD remains unclear, a better understanding of IDD pathogenesis could provide a better treatment for IDD (Feng et al., 2016). The pathogenesis of IDD mainly includes trauma, oxidative stress, genetic susceptibility, and aging, which is rarely investigated. Cellular senescence, a permanent state of cell cycle arrest, plays a possible role in the pathogenesis of age-associated IDD (Wang et al., 2016). Currently, cellular senescence is associated with age-related, microenvironment-derived stresses that may lead to accelerated disc degeneration. On the other hand, the IVD consists of the nucleus pulposus, annulus fibrosus, and endplate. It has been determined that senescence of nucleus pulposus cells and annulus fibrosus cells plays a significant role in IDD (Chen et al., 2016; Gao et al., 2018). However, the effect of senescent cells in endplate subchondral bone on IDD has not been clarified.

It is well-known that the nucleus pulposus and two thirds of the annulus fibrosus are avascular, whose nutrient supply relies on molecular diffusion from capillaries in the sub-endplate region (Yuan et al., 2015). With increasing age, the senescence of vascular ECs and osteoblasts in the marrow space of endplate may be related to the decrease of bone mass and capillaries in sub-endplate, which inevitably leads to the reduced nutrient supply of IVD. Therefore, we propose that the use of senolytics to eliminate senescent cells in the marrow space of the bony endplate could improve the coupling of angiogenesis and osteogenesis, and greatly improve the nutrient supply of IVD. In this study, we looked at the degenerative change of IVD in 18-month-old mice (Veh and D + Q), and in the same way, 3-month-old mice (Ctr) were designed as a point of reference. In our aged mice model, the vertebral bone mass and IVD height loss that worsened with age was linked to the development of IDD, as validated by various research (Khosla et al., 2018; Kague et al., 2021). While most previous studies on senolytics targeted senescent cells in the nucleus pulposus and annulus fibrosus, our study creatively put forward that senolytics could improve IVD blood supply by elimination of senescent cells in the marrow space of the bony endplate, further alleviate IDD. Indeed, due to the absence of blood vessels in the nucleus pulposus and inner two-third annulus fibrosus, many drugs with larger molecular weights are difficult to reach the target site, and previous studies have not confirmed whether senolytics can directly enter the nucleus pulposus or not. In this study, it is more persuasive that senolytics can directly reach the sub-endplate region with abundant microvessels. In addition, senolytics can indirectly play an anti-senescence role by improving the nutrient supply of IVD and promoting energy metabolism of NP and AF cells, even if they cannot reach the IVD directly to clear senescent nucleus pulposus cells and annulus fibrosus cells. Therefore, the treatment for IDD by elimination of senescent cells in the marrow space of the bony endplate is sufficiently scientific and feasible.

However, this study is still not perfect in view of the following aspects. First, because our study only had one observation time point, it is still unclear whether the benefits reported are restorative or preventive, and further in vivo research that focus on the middle and later stages of the IDD are needed. Second, in this investigation, we exclusively used female mice to decrease animal individual differences. Although it is still uncertain if gender has a role in disc degeneration, the results of this study are now confined to females. Third, in terms of imaging, only Micro-CT was used to evaluate the degeneration of the disc, while MR with T1ρ or T2map sequences could more comprehensively evaluate the quantitative changes of the extracellular matrix of the NP during disc degeneration and regeneration, which should be applied in our future studies. Finally, although the increase of microvessels in sub-endplate is persuasive in the causal relationship between improved nutrient supply and energy metabolism of the IVD, we did not directly observe the corresponding changes within the IVD. In future studies, we will adopt more advanced research methods, for example, quantitative detection of energy metabolism changes of IVDs, especially nucleus pulposus cells, by 18F-FDG combined with micro-PET-MR. Nonetheless, to our knowledge, this study is the first to demonstrate the effectiveness of D + Q in eliminating senescent cells in the marrow space of the bony endplate, promoting the increase of bone mass and capillary density in endplate subchondral marrow, and thus alleviating IDD. Future research, we anticipate, will continue to conduct in-depth research in this field and provide more reliable evidence for the therapeutic use of senolytics in clinical treatment of IDD.

## Data Availability

The original contributions presented in the study are included in the article/Supplementary Material; further inquiries can be directed to the corresponding authors.
